# A Bloom's Taxonomy-integrated rotation model enhances clinical reasoning and practical skills in optometry interns during ophthalmology rotation: a randomized controlled trial

**DOI:** 10.3389/fmed.2026.1746533

**Published:** 2026-02-02

**Authors:** Yi Cui, Li Wang, Chenghuan Dong, Huiqin Cheng, Desheng Fu, Jianzhang Hu, Yan Huang

**Affiliations:** 1 Department of Optometry, School of Medical Technology and Engineering, Fujian Medical University, Fuzhou, Fujian, China; 2 Department of Ophthalmology, Fujian Medical University Union Hospital, Fuzhou, Fujian, China

**Keywords:** Bloom's Taxonomy, clinical optometry interns, competency-based education, formative assessment, student-mentor rotation system

## Abstract

Traditional clinical medical internship models often lack structured educational objectives and comprehensive assessments, limiting holistic competency development. This study evaluates the effectiveness of a novel internship education management model integrating Bloom's Taxonomy with a student-mentor rotation system to enhance cognitive, affective, and psychomotor skills. We conducted a randomized controlled trial with 54 optometry interns during their ophthalmology clinical rotation, assigning them to either an experimental group (*n* = 30) using the Bloom's Taxonomy-integrated rotation model or a control group (*n* = 24) receiving traditional training. Both groups completed 12-week rotations with identical duration and content. The Bloom's Taxonomy-integrated rotation model was organized into three progressive phases corresponding to ascending levels of Bloom's Taxonomy (knowledge acquisition → clinical application → synthesis). Outcomes were assessed through written examinations, practical skill test, and course experience questionnaires. The Bloom's Taxonomy-integrated rotation group showed superior performance in case analysis, history taking and practical skills. They also reported significantly higher satisfaction in problem-solving, motivation and feedback quality. In conclusion, the Bloom's Taxonomy-integrated rotation model demonstrated superior clinical reasoning, practical skills, and medical interns' satisfaction, among optometry interns compared to traditional training. The Bloom's Taxonomy-integrated rotation model represents an effective, structured framework for enhancing medical internship training, though warranting further validation in broader settings.

## 1 Introduction

The current education management models for clinical medical internships exhibit numerous shortcomings in practical teaching and require urgent improvement. Firstly, educational objectives often lack a hierarchical structure, primarily focusing on basic clinical operations or observations while neglecting higher-order cognitive skills, affective goals, and comprehensive skill development (1). For instance, during surgical rotations, students are frequently limited to passive observation, with insufficient opportunities for in-depth analysis and reflection, hindering their holistic development. Secondly, the evaluation methods require optimization. Although existing assessment systems have begun incorporating key metrics such as students' performance in case discussions and doctor-patient communication skills, these critical competencies remain underrepresented in overall evaluations (2). Further refinement of assessment dimensions, such as clinical reasoning, integrative analytical ability, and teamwork, would be beneficial. Additionally, the evaluation of doctor-patient communication skills should adopt a more comprehensive approach, encompassing communication techniques, patient education, and responses to complex scenarios (3). By increasing the weight of these indicators in assessments and employing standardized evaluation tools and multidimensional feedback mechanisms, the holistic development of students' competencies can be better promoted. Introducing more scientifically grounded educational frameworks is therefore essential.

In many clinical teaching hospitals in our setting, the traditional ophthalmology internship follows a standard department-based rotation plan, where students are typically assigned to a single supervising mentor and rotate through core subspecialties primarily for observational learning. This model often lacks structured objectives, phased progression, and systematic feedback, thereby limiting the consistent development of higher-order clinical reasoning, integrated practical skills, and professional competencies among interns.

The “student-mentor rotation system,” a flexible and systematic education management model, has garnered increasing attention in medical education in recent years. This model emphasizes personalized guidance and holistic development through collaboration and interaction between students and multiple mentors, aiming to enhance students' practical skills and overall competencies (4). Theoretically, the rotation system combined with the mentorship model underscores “personalization” and “continuity.” Personalization is reflected in tailored learning plans based on students' individual learning needs and abilities, while continuity is achieved through phased rotations, allowing students to engage comprehensively with clinical practice under the guidance of different mentors in various departments (5). Studies have shown that multidisciplinary rotations enable students to acquire a more comprehensive knowledge base and foster interdisciplinary integration skills (6, 7). Implementing the rotation system requires a clear definition of mentors' roles and responsibilities, well-organized rotation schedules, and a strong emphasis on feedback mechanisms. Mentors are responsible not only for clinical teaching but also for supporting students' career planning and psychological wellbeing. This comprehensive guidance significantly enhances students' practical skills and professional competencies (8). By setting clear rotation schedules and task objectives, students can progressively develop their capabilities, from mastering basic operations to handling complex diagnostic and therapeutic procedures. Research indicates that such systematic arrangements effectively improve students' comprehensive abilities in disease management (9, 10). Additionally, regular feedback sessions help students identify and address learning challenges promptly. Studies have demonstrated a positive correlation between the frequency of feedback and improvements in students' clinical skills (11). By incorporating multi-mentor guidance and phased rotation arrangements, this model can comprehensively enhance students' clinical competencies and professional development, providing both theoretical and practical foundations for optimizing internship education management.

Bloom's Taxonomy, has been widely applied across various educational levels, providing a clear framework for formulating and evaluating educational objectives (12). In medical education, this taxonomy refines educational goals into three domains: cognitive, affective, and psychomotor, facilitating the comprehensive development of students' theoretical knowledge, practical skills, and professional qualities (13). The cognitive domain encompasses six hierarchical levels: remembering, understanding, applying, analyzing, evaluating, and creating. Cognitive objectives in medical education, such as mastering fundamental medical knowledge, clinical diagnostic methods, and therapeutic strategies, help students transition from foundational knowledge acquisition to the cultivation of advanced clinical reasoning skills (14, 15). The affective domain focuses on nurturing students' attitudes, values, and professionalism, with particular emphasis on developing doctor-patient communication skills, empathy, and ethical conduct in medical education (16). The psychomotor domain emphasizes the development of skills ranging from basic motor tasks to complex professional techniques. Research has revealed that the application of Bloom's Taxonomy in medical education significantly enhances students' clinical decision-making abilities and overall competencies (17). By incorporating comprehensive assessment indicators across cognitive, affective, and psychomotor domains, Bloom's Taxonomy provides a more accurate reflection of students' learning outcomes and optimizes teaching effectiveness (18). This taxonomy offers a scientific foundation for setting and evaluating educational objectives in medical education. However, despite its recognized value, a significant gap remains in how to systematically operationalize Bloom's Taxonomy within the practical constraints of a structured clinical rotation. What we need is a translational educational model that integrates its cognitive hierarchy with real clinical mentorship and a time-bound rotation schedule.

This study aimed to develop and evaluate a novel internship education management model that explicitly embeds Bloom's Taxonomy within a student-mentor rotation system. We hypothesized that this integrated approach would provide a clear, phased learning trace, enhancing not only knowledge, more critically, the higher-order competencies of clinical reasoning, practice skill, and professional communication, thereby offering a replicable framework for competency-based clinical education.

## 2 Materials and methods

### 2.1 Participants

The study participants consisted of 54 clinical medical interns from Fujian Medical University, all from the same ophthalmology and optometry class. All had uniformly completed the same structured curriculum in basic medical and optical sciences prior to the clinical internship. They were randomly assigned to two groups: the experimental group (Bloom's Taxonomy-integrated rotation model) and the control group (traditional internship education management model). Randomization was performed using a computer-generated random number sequence with a 1:1 allocation ratio. The allocation sequence was generated by an independent researcher not involved in participant enrollment or teaching. After randomization but prior to the commencement of the study intervention, six participants allocated to the control group were unable to participate due to unforeseen and study-unrelated reasons (specifically, personal leave or unforeseen transfers to other departments). Consequently, the final analysis included 30 participants in the experimental group and 24 in the control group. Analysis was performed on data from these study completers. A reassessment of baseline characteristics, including age, gender, and prior ophthalmology scores, were comparable between groups (Table 1), confirming that post-randomization attrition did not undermine the initial randomization.

**Table 1:** Characteristics of the participants of the two groups.

**Characteristics**	**Experimental group (*n* = 30)**	**Control group (*n* = 24)**	**χ^2^/t**	***P- value***
**Sex**				
Male, *n* (%)	14 (46.67)	13 (54.17)	1.63	0.20
Female, *n* (%)	16 (53.3)	11 (45.83)		
Age in years (mean ± SD)	22.93 ± 0.68	23.04 ± 0.68	2.00	0.57
Ophthalmology theoretical examination score (mean ± SD)	80.45 ± 8.02	81.00 ± 8.63	2.01	0.82

The inclusion criteria: 2022 cohort optometry interns who had completed prior basic medical courses and a 12-week (3-month) clinical ophthalmology internship. And the exclusion criteria were students who, after enrollment, withdrew or were absent for a prolonged period not related to the study intervention, thus failing to complete the internship protocol. The two groups were comparable, and all students were aware of and agreed to the distribution scheme for the teaching methods. This study received approval by the Ethics Committee of the Fujian Medical University Union Hospital ethics committee (2022KY132), and executed in accordance with the declaration of Helsinki. Written informed consent was obtained from all participants prior to enrollment by the study coordinator.

### 2.2 Study intervention design

The experimental group adopted a structured education model based on Bloom's Taxonomy, incorporating student-mentor rotation system to design a systematic ophthalmology internship rotation plan. Students rotated through four core subspecialties: ocular surface disease, cataract and glaucoma, vitreoretinopathy, and general ophthalmology, with each rotation lasting 2–3 weeks under the guidance of designated subspecialty mentors. Each subspecialty week was supervised by a mentor who served as both clinical instructor and evaluator. The 12-week program was organized into three progressive phases (early, mid, late) corresponding to ascending cognitive domain of Bloom's Taxonomy (knowledge acquisition → clinical application → synthesis/evaluation). Mentors tailored the learning content and tasks for each rotation phase according to these taxonomic levels, ensuring a graduated development of clinical competencies. We employ novel teaching methods tailored to the instructional content throughout the program (19, 20).

In the early phase (weeks 1–4), knowledge was built through interactive lectures on core ophthalmic principles, virtual patient simulation for pattern recognition and diagnostic reasoning, and structured mentor-led case discussions that reinforced foundational recall and comprehension. In the mid phase (weeks 5–8), training shifted to supervised hands-on practice following a “watch-one, do-one, teach-one” framework. Students performed key procedures, including slit-lamp examination, fundoscopy, and tonometry, using standardized checklists, while mentors provided immediate feedback on both psychomotor skill execution and the accompanying clinical decision-making. In the late phase (weeks 9–12), learners demonstrated independent competence by completing comprehensive case reports based on multi-morbidity simulated or de-identified real patient records. These reports required a full workup, differential diagnosis, evidence-based management plan, and follow-up strategy, which students then defended in a mini-case presentation, thereby engaging in higher-order synthesis, evaluation, and justification of clinical reasoning. After rotation, mentors provided structured feedback at each phase using a set of standardized assessment tools, which included procedure-specific checklists for clinical skills, structured feedback forms aligned with Bloom's Taxonomy levels for each phase, and final integrated reports synthesized performance data with personalized improvement recommendations. This constituted a structured, phase-specific formative feedback system. The feedback content was explicitly aligned with the cognitive objectives of each Bloom's Taxonomy level and was delivered continuously through the multi-mentor rotation, ensuring personalized and developmental guidance.

The control group interns follow a standard and traditional department rotation management plan, supervised psychomotor skill execution throughout by a single assigned mentor. Although they rotated through the same four subspecialties, their training lacked the Bloom's Taxonomy framework and multi-mentor rotation system. Without phased objectives or structured feedback, learning was primarily observational and task-based, focusing on routine clinical assistance and informal case discussions. Any feedback provided was *ad-hoc*, informal, and not systematized around progressive learning objectives. Both groups of interns will complete a three-month clinical internship.

### 2.3 Evaluation

The evaluation in this study was conducted through written exam scores, clinical practice skill assessments, and the course experience questionnaire (CEQ) (21). The written exam comprised two sections, which included Theoretical Foundations and Case-Based Analysis, and was designed to measure students' knowledge mastery in the cognitive domain. Clinical practice skill assessments evaluated students' skill level in actual procedures, including History Taking, Case Analysis, Physical Examination, and Professionalism, corresponding to the psychomotor domain, using standardized checklists developed based on the competency standards of the national standardized residency training program. To further ensure consistency, all mentors were trained on these phase-specific objectives and used uniform assessment, thereby minimizing potential scoring variability across subspecialties. The CEQ was administered using a 5-point Likert scale (1 = Strongly Disagree to 5 = Strongly Agree) to assess the learning experience and satisfaction in the affective domain through students' feedback on the teaching process. This evaluation provided quantitative evidence for the effectiveness of the novel Bloom's Taxonomy-integrated rotation model.

### 2.4 Statistical analysis

All statistical analyses were performed using GraphPad Prism. Continuous variables, including age, prior and post-intervention exam scores, and all CEQ scores, are presented as means ± SD. The normality of these continuous data was verified using the Shapiro-Wilk test (*P* > 0.05) and visual inspection of Q-Q plots. All satisfied the normality assumption. Therefore, between-group comparisons for these continuous outcomes were analyzed using independent samples *t*-tests. Additionally, effect sizes (Cohen's d) and their 95% confidence intervals were reported. The categorical variable, sex, was compared between groups using the Chi-square test. Statistical significance was defined as *P* < 0.05.

## 3 Results

The basic characteristics of the two groups are shown in Table 1. The experimental group (Bloom's Taxonomy-integrated rotation model) consisted of 30 participants, with 16 females and 14 males, and an average age of 22.93 years. The control group (traditional internship model) consisted of 24 participants, with 11 females and 13 males, and an average age of 23.04 years. There were no significant differences between the two groups in terms of gender (assessed by the Chi-square test), age, or previous ophthalmology exam scores (assessed by independent samples *t*-tests) (*P* > 0.05).

Then, we compared the students' perceptions of the experimental group and the control group in Table 2 (CEQ score, 5-point Likert scale). More students in the experimental group reported that it was more helpful in developing their problem-solving abilities (3.86 ± 0.61 vs. 3.28 ± 0.81, *P* < 0.01) and analytical skills (3.65 ± 0.62 vs. 3.03 ± 0.57, *P* < 0.001), enhancing knowledge memory retention (3.68 ± 0.59 vs. 3.16 ± 0.57, *P* < 0.01), and motivating learning (3.89 ± 0.49 vs. 3.02 ± 0.67, *P* < 0.0001). More students in the experimental group (3.91 ± 0.48 vs. 3.15 ± 0.81, *P* < 0.001) felt they understood the expected work standards. There were no significant differences between the two groups in terms of learning pressure; however, more students in the control group felt the pre-class assignments were too heavy (2.61 ± 0.72 vs. 3.02 ± 0.67, *P* = 0.04). In addition, more students in the experimental group felt capable of solving unfamiliar problems (3.76 ± 0.49 vs. 3.10 ± 0.70, *P* < 0.001), and also felt that the teachers provided more and quicker feedback (3.68 ± 0.64 vs. 3.02 ± 0.67, *P* < 0.001). Overall, the satisfaction with the internship program in the experimental group was significantly higher than that in the control group (3.82 ± 0.74 vs. 3.05 ± 0.66, *P* < 0.001).

**Table 2:** Comparison of the modified course experience questionnaire between the two groups.

**Questions**	**Experimental group**	**Control group**	***t***	***P-value***	**Cohen's d [95% CI]**
It is always easy here to know the standard of work expected	3.91 ± 0.48	3.15 ± 0.81	4.17	0.00	1.10 [0.56, 1.63]
The course developed my problem-solving skills	3.86 ± 0.61	3.28 ± 0.81	2.91	0.01	0.79 [0.27, 1.30]
The course sharpened my analytic skills	3.65 ± 0.62	3.03 ± 0.57	3.70	0.00	1.03 [0.50, 1.55]
The course promotes the memorization of knowledge	3.68 ± 0.59	3.16 ± 0.57	3.18	0.00	0.89 [0.37, 1.40]
The course helps enhance my motivation to learn	3.89 ± 0.49	3.02 ± 0.67	5.43	0.00	1.51 [0.96, 2.05]
The course gives me too much pre-class work	2.61 ± 0.72	3.02 ± 0.67	2.11	0.04	−0.59 [−1.09, −0.08]
Teaching staff here normally give helpful feedback on how you are doing	3.68 ± 0.64	3.02 ± 0.67	3.67	0.00	1.00 [0.48, 1.52]
There are many pressures on me to do well in this course	2.23 ± 0.78	2.43 ± 0.66	0.96	0.34	−0.27 [−0.76, 0.22]
I feel confident about tackling unfamiliar problems	3.76 ± 0.49	3.10 ± 0.70	3.97	0.00	1.09 [0.55, 1.62]
Overall, I am satisfied with the quality of this course	3.82 ± 0.74	3.05 ± 0.66	3.88	0.00	1.07 [0.53, 1.60]

**Figure 1:**
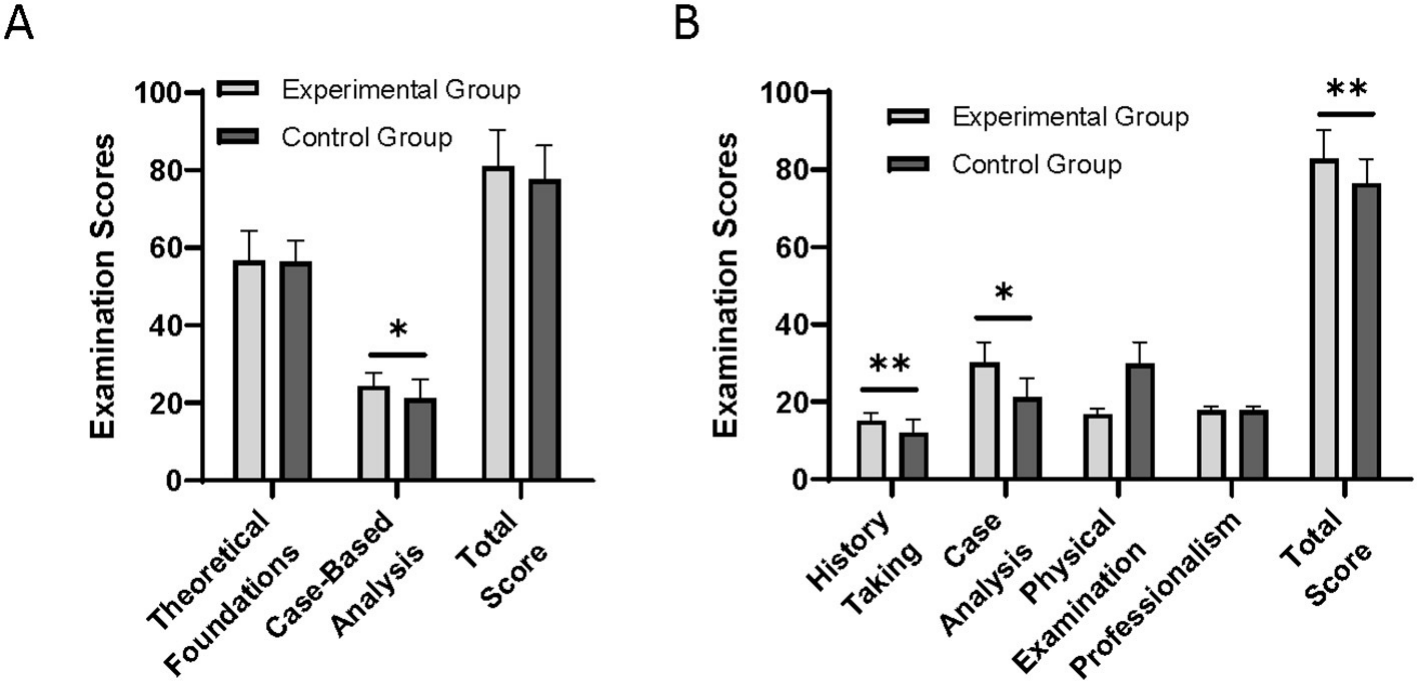
Comparison of **(A)** written examination and **(B)** practical clinical skills examination between the experimental group and the control group. Data represent mean ± SD and analyzed by unpaired student's *t*-test. *P < 0.05, **P < 0.01.

As shown in Figure 1, the total practical clinical skills exam scores in the experimental group were significantly higher than those in the control group (82.70 ± 7.40 vs. 76.42 ± 6.34, *P* < 0.01). In both the written exam and the practical skills exam, the experimental group outperformed the control group in case analysis (24.33 ± 3.36 vs. 21.21 ± 4.84, *P* < 0.05; 33.03 ± 5.41 vs. 29.83 ± 5.61, *P* < 0.05). Notably, in the medical history taking section, a core competency within the affective domain that emphasizes communication, the experimental group's performance was significantly better than that of the control group (15.10 ± 1.99 vs. 12.04 ± 3.41, *P* < 0.001). Improvements were also observed in other domains with affective components, such as professionalism. There was no significant difference between the two groups in terms of theoretical foundation scores (56.63 ± 7.77 vs. 56.38 ± 5.50).

## 4 Discussion

This study, based on Bloom's Taxonomy of educational objectives and the student-mentor rotation system, developed an innovative educational management model for clinical optometry interns and experimentally validated its effectiveness. Bloom's Taxonomy providing a structured framework designed to create a systematic and hierarchical learning path, which our results suggest was effective in enhancing specific competencies in students' practical clinical skills, cognitive abilities, doctor-patient communication, and other comprehensive qualities. Furthermore, the introduction of the student-mentor rotation system achieved personalized guidance and comprehensive development of educational objectives. Through collaboration and interaction between students and multiple mentors, this system addressed the uneven mentorship issue present in traditional models and optimized the allocation of teaching resources. The study indicated that this model was associated with enhanced bidirectional feedback and improvements in students' clinical practice abilities, analytical and clinical reasoning skills, and professional qualities.

The enhanced performance observed in the experimental group may be explained through the underlying educational principles embedded in the integrated model. Bloom's Taxonomy provided a structured cognitive scaffold that systematically decomposed clinical competence into sequential, manageable levels. This hierarchical approach likely reduced cognitive load by allowing students to solidify basic knowledge and skills before progressing to complex integration and evaluation tasks, thereby facilitating deeper learning. The design of the rotation phases incorporated the principle of deliberate practice, as each phase set clear objectives and enabled repeated, mentored execution of skills tailored to a specific cognitive level. Concurrently, the student-mentor rotation system established a continuous formative feedback loop. Mentors, aligned with the phase-specific objectives, provided timely and targeted feedback—ranging from reinforcement of core knowledge in early stages to refinement of clinical reasoning in later stages. This alignment between feedback content and the learner's cognitive stage is known to enhance skill acquisition and development. Thus, the effectiveness of the model likely stems from the synergy between a clear, hierarchical learning pathway, and an adaptive, feedback-rich mentoring environment, both of which are grounded in established theories of cognitive and skill development.

The student-mentor rotation system ensures that each student, under the guidance of multiple mentors, is exposed to a wide range of clinical issues and teaching methods. This rotation arrangement not only aids in enhancing students' knowledge and skills across different fields but also promotes the development of their overall qualities. At the end of each rotation, mentors conduct comprehensive evaluations of students, assessing their progress in the cognitive, emotional, and skill domains, and adjust teaching strategies based on the evaluation results. During the rotation process, mentors provide personalized learning feedback and guidance through interactions with students, ensuring that they achieve the expected educational goals within the framework of Bloom's Taxonomy.

This study demonstrated that integrating Bloom's Taxonomy with the student-mentor rotation model substantially improved the overall competencies and practical outcomes of clinical medical interns, offering valuable theoretical insights and practical guidance for the enhancement of medical education management. Our results indicated that the clarity and systematization of Bloom's Taxonomy effectively improved students' higher cognitive abilities and skills in practice, as evidenced by their superior performance in case analysis, which aligns with studies demonstrating its efficacy in developing clinical reasoning skills (14, 22). Furthermore, the significant gains in clinical communication reflect the taxonomy's structured approach to affective domain training (23), while the increased learner motivation reported in the CEQ are consistent with findings that competency-based frameworks enhance engagement (24, 25). This conclusion aligns with existing research, which shows that the hierarchical structure of Bloom's Taxonomy can effectively facilitate the learning process, fostering the development of both basic skills and critical thinking (12, 26). It is noteworthy that the higher standard deviation in the control group's history-taking scores suggests greater variability in their performance, potentially reflecting the lack of structured training in this area within the traditional model.

Interestingly, while the Bloom's Taxonomy-integrated rotation model significantly enhanced clinical reasoning, history-taking, and practical skills, it did not confer an advantage in theoretical written exam scores over the traditional model. This finding likely reflects a fundamental distinction between knowledge recall and knowledge application. The theoretical exam primarily assessed foundational knowledge, which was covered comparably in both groups. In contrast, the experimental model explicitly targeted higher-order cognitive domains, as reflected in the superior performance in case analysis and clinical skills. This also points to the limited capacity of written examinations alone to capture the full spectrum of clinical competency emphasized in modern medical education. In addition, it is noted that the control group scored slightly higher on the “physical examination” sub-item. This may reflect random variation given the sample size, and more importantly, a divergent learning focus. The traditional model's emphasis on observational, task-specific practice may lead to better performance on isolated procedural checklists. In contrast, our model prioritizes the integration of skills with diagnostic reasoning, which are reflected in superior case analysis and overall practical scores.

It is worth mentioning that the experimental group stands out particularly in the cultivation of doctor-patient communication skills, specifically in the area of medical history taking. The traditional model often neglects systematic training of these abilities, while the Bloom's Taxonomy, with its clear emotional goals, incorporates this into the teaching design. Combined with the real-time guidance provided by mentors during the rotation system, it significantly enhanced students' performance in actual clinical situations. This outcome supports the effectiveness of a teaching model that combines emotional and skill goals in the comprehensive training of medical clinical internship. Furthermore, the experimental group's superior performance in objectively assessed affective competencies, specifically in history-taking and professionalism domains of the clinical skills exam, which aligns with their higher self-reported ratings on the CEQ regarding communication confidence and perceived feedback quality. This convergence of behavioral assessment and learner perception underscores that the integrated model effectively promoted the internalization and behavioral expression of targeted affective domain objectives.

In addition, some unexpected results were observed in the study, such as the achievement in psychomotor skill objectives (notably history-taking) being more pronounced than that in higher-order cognitive integration objectives. This discrepancy may be explained through cognitive load theory (27, 28) and the direct feedback mechanism embedded in the practical training. The structured skill practice with immediate feedback reduces extraneous cognitive load and accelerates procedural learning. In contrast, applying knowledge in new clinical contexts is more cognitively demanding and requires prolonged, repeated practice to master. This finding provides insights for the improvement of future teaching models, suggesting the need to balance the proportion of theoretical learning and practical operations in instructional design, ensuring that students achieve a balanced development between skill and cognitive goals.

This study has the following limitations. First, the relatively small sample size and restriction to a single ophthalmology teaching center significantly limit the generalizability of our findings. As a pilot study, no sample size calculation was performed, the cohort was based on available trainees. Further research is required to evaluate the model's applicability across diverse clinical disciplines with varying demands and operational environments, and future studies should incorporate larger, multi-center cohorts with formal sample size estimation to strengthen external validity. Second, the 12-week duration assessed only short-term outcomes. The long-term impact on higher-order cognitive abilities, professional development, clinical independence, and career adaptability remains unknown and necessitates longitudinal follow-up. Third, the study design did not include repeated pre- and post-intervention assessments within each group. Consequently, we are unable to analyze the trajectory or magnitude of competency development specifically attributable to each training model over the internship period. Fourth, the lack of blinding of clinical skills examiners is a potential limitation, although standardized checklists and a multi-rater assessment design were employed to mitigate assessment bias. Furthermore, the CEQ provided a broad measure of student satisfaction and perceived learning climate but was not designed to assess the hierarchical levels of the affective domain. While established summative exams provided a standardized comparative endpoint, the structured formative assessment system was implemented only within the experimental group as an integral component of the educational intervention. Future studies may introduce comparable formative assessments in both arms to allow direct comparison of learning processes alongside final competency outcomes. Fifth, widespread implementation of this model to multiple specialties faces resource allocation challenges in clinical teaching hospitals. Variations in case complexity, mentor availability, and equipment resources among different disciplines may impede the attainment of uniform educational objectives. Therefore, optimizing teaching resource allocation strategies and balancing the demands of teaching and medical services will be an important challenge for future expansion. Additionally, the fixed rotation duration might be insufficient to fully achieve all complex learning objectives, particularly in certain subspecialties. Future adaptations could consider competency-based progression.

In conclusion, this study demonstrates that integrating Bloom's Taxonomy with a structured student-mentor rotation system significantly enhanced clinical reasoning, practical skills, and satisfaction among optometry interns compared to traditional training. The findings support the effectiveness of this combined framework for achieving comprehensive educational objectives within the specific context of ophthalmology internship. It offers valuable insights for educational reforms in other professional fields and holds reference value for clinical medical education both domestically and internationally.

## Data Availability

The original contributions presented in the study are included in the article/Supplementary material, further inquiries can be directed to the corresponding authors.
