# Supplementary material for: A Bloom's Taxonomy-integrated rotation model enhances clinical reasoning and practical skills in optometry interns during ophthalmology rotation: a randomized controlled trial
Source: Front Med (Lausanne). 2026 Feb 2;13:1746533. doi: 10.3389/fmed.2026.1746533 (PMC12907170; doi:10.3389/fmed.2026.1746533)
Supplement: Supplementary file 1 [file Data_Sheet_1.docx]

Supplementary Material

# Supplementary Figures and Tables

## Supplementary Tables

**Supplementary Table 1.** Baseline characteristics of the participants.

| ID | Group | Gender | Age | Ophthalmology theoretical examination score |
| --- | --- | --- | --- | --- |
| 1 | Experimental | Male | 23 | 89.2 |
| 2 | Control | Male | 23 | 79 |
| 3 | Control | Female | 23 | 60.4 |
| 4 | Control | Male | 22 | 85 |
| 5 | Experimental | Male | 23 | 72 |
| 6 | Experimental | Female | 23 | 81 |
| 7 | Experimental | Female | 23 | 67 |
| 8 | Experimental | Female | 24 | 67 |
| 9 | Control | Female | 23 | 89.8 |
| 10 | Control | Male | 23 | 94.6 |
| 11 | Experimental | Male | 23 | 85.4 |
| 12 | Experimental | Female | 23 | 89 |
| 13 | Experimental | Female | 23 | 89 |
| 14 | Control | Female | 24 | 84.6 |
| 15 | Control | Male | 22 | 81.6 |
| 16 | Control | Female | 23 | 74.6 |
| 17 | Experimental | Male | 23 | 65 |
| 18 | Experimental | Male | 23 | 75 |
| 19 | Control | Female | 23 | 77 |
| 20 | Control | Male | 22 | 72 |
| 21 | Experimental | Male | 22 | 89 |
| 22 | Experimental | Female | 22 | 80.2 |
| 23 | Control | Male | 23 | 84.4 |
| 24 | Experimental | Male | 22 | 87.8 |
| 25 | Control | Female | 23 | 90.4 |
| 26 | Experimental | Female | 22 | 84.2 |
| 27 | Control | Male | 24 | 82.4 |
| 28 | Experimental | Female | 24 | 76 |
| 29 | Experimental | Male | 22 | 69 |
| 30 | Experimental | Male | 24 | 93.6 |
| 31 | Control | Female | 22 | 84.4 |
| 32 | Experimental | Female | 22 | 92.2 |
| 33 | Experimental | Female | 23 | 88 |
| 34 | Experimental | Male | 23 | 74 |
| 35 | Control | Female | 24 | 69 |
| 36 | Experimental | Male | 23 | 83.4 |
| 37 | Control | Male | 23 | 74 |
| 38 | Control | Female | 24 | 73 |
| 39 | Experimental | Female | 23 | 74 |
| 40 | Control | Male | 24 | 90 |
| 41 | Experimental | Female | 23 | 78 |
| 42 | Experimental | Male | 23 | 73 |
| 43 | Control | Male | 23 | 77 |
| 44 | Experimental | Female | 22 | 79.6 |
| 45 | Experimental | Male | 23 | 74 |
| 46 | Control | Female | 22 | 75 |
| 47 | Control | Male | 23 | 72 |
| 48 | Experimental | Female | 24 | 89 |
| 49 | Control | Male | 23 | 91 |
| 50 | Experimental | Female | 24 | 83 |
| 51 | Experimental | Female | 24 | 87 |
| 52 | Control | Female | 22 | 87.6 |
| 53 | Experimental | Female | 22 | 79 |
| 54 | Control | Female | 24 | 95.2 |

**Supplementary Table 2.** Raw scores of the written examination for both the control and experimental groups.

| Experimental group | | | Control group | | |
| --- | --- | --- | --- | --- | --- |
| Theoretical foundations | Case-based analysis | Total score | Theoretical foundations | Case-based analysis | Total score |
| 56 | 28 | 84 | 56 | 28 | 84 |
| 54 | 19 | 73 | 54 | 19 | 73 |
| 59 | 25 | 84 | 59 | 25 | 84 |
| 57 | 16 | 73 | 57 | 16 | 73 |
| 50 | 15 | 65 | 50 | 15 | 65 |
| 51 | 13 | 64 | 51 | 13 | 64 |
| 62 | 23 | 85 | 62 | 23 | 85 |
| 59 | 15 | 74 | 59 | 15 | 74 |
| 47 | 16 | 63 | 47 | 16 | 63 |
| 50 | 25 | 75 | 50 | 25 | 75 |
| 63 | 25 | 88 | 63 | 25 | 88 |
| 58 | 24 | 82 | 58 | 24 | 82 |
| 65 | 25 | 90 | 65 | 25 | 90 |
| 47 | 24 | 71 | 47 | 24 | 71 |
| 55 | 27 | 82 | 55 | 27 | 82 |
| 66 | 27 | 93 | 66 | 27 | 93 |
| 49 | 19 | 68 | 49 | 19 | 68 |
| 58 | 24 | 82 | 58 | 24 | 82 |
| 53 | 10 | 63 | 53 | 10 | 63 |
| 60 | 24 | 84 | 60 | 24 | 84 |
| 65 | 20 | 85 | 65 | 20 | 85 |
| 58 | 24 | 82 | 58 | 24 | 82 |
| 58 | 23 | 81 | 58 | 23 | 81 |
| 53 | 18 | 71 | 53 | 18 | 71 |
|  |  |  | 57 | 25 | 82 |
|  |  |  | 56 | 20 | 76 |
|  |  |  | 45 | 17 | 62 |
|  |  |  | 56 | 28 | 84 |
|  |  |  | 63 | 19 | 82 |
|  |  |  | 56 | 27 | 83 |

**Supplementary Table 3.** Raw scores of the practical clinical skills examination for both the control and experimental groups.

| Control group | | | | | Experimental group | | | | |
| --- | --- | --- | --- | --- | --- | --- | --- | --- | --- |
| History taking | Case analysis | Physical examination | Professionalism | Total score | History taking | Case analysis | Physical examination | Professionalism | Total score |
| 9 | 39 | 17 | 17 | 82 | 15 | 28 | 15 | 17 | 75 |
| 16 | 35 | 17 | 19 | 87 | 16 | 32 | 16 | 18 | 82 |
| 12 | 29 | 15 | 18 | 74 | 16 | 37 | 15 | 17 | 85 |
| 16 | 35 | 17 | 19 | 87 | 18 | 38 | 18 | 19 | 93 |
| 13 | 20 | 17 | 18 | 68 | 16 | 27 | 17 | 18 | 78 |
| 12 | 24 | 16 | 17 | 69 | 15 | 25 | 18 | 17 | 75 |
| 17 | 31 | 18 | 19 | 85 | 14 | 38 | 17 | 19 | 88 |
| 13 | 33 | 18 | 18 | 82 | 16 | 31 | 18 | 18 | 83 |
| 18 | 18 | 17 | 17 | 70 | 16 | 35 | 14 | 17 | 82 |
| 11 | 27 | 15 | 19 | 72 | 19 | 35 | 18 | 20 | 92 |
| 12 | 25 | 15 | 17 | 69 | 10 | 30 | 15 | 17 | 72 |
| 13 | 25 | 16 | 18 | 72 | 14 | 33 | 18 | 19 | 84 |
| 11 | 23 | 17 | 19 | 70 | 16 | 30 | 16 | 16 | 78 |
| 16 | 37 | 17 | 16 | 86 | 13 | 37 | 15 | 17 | 82 |
| 9 | 31 | 17 | 17 | 74 | 18 | 39 | 19 | 18 | 94 |
| 15 | 30 | 17 | 18 | 80 | 16 | 40 | 18 | 19 | 93 |
| 6 | 29 | 16 | 19 | 70 | 10 | 28 | 16 | 16 | 70 |
| 8 | 28 | 17 | 18 | 71 | 13 | 35 | 17 | 17 | 82 |
| 10 | 36 | 18 | 17 | 81 | 18 | 37 | 18 | 20 | 93 |
| 7 | 30 | 17 | 16 | 70 | 15 | 21 | 15 | 17 | 68 |
| 17 | 25 | 16 | 17 | 75 | 15 | 32 | 17 | 18 | 82 |
| 8 | 35 | 17 | 18 | 78 | 16 | 27 | 18 | 19 | 80 |
| 8 | 39 | 19 | 17 | 83 | 15 | 35 | 19 | 18 | 87 |
| 12 | 32 | 17 | 18 | 79 | 14 | 43 | 19 | 17 | 93 |
|  |  |  |  |  | 13 | 26 | 15 | 19 | 73 |
|  |  |  |  |  | 14 | 40 | 18 | 18 | 90 |
|  |  |  |  |  | 15 | 40 | 16 | 16 | 87 |
|  |  |  |  |  | 15 | 25 | 15 | 18 | 73 |
|  |  |  |  |  | 16 | 31 | 16 | 17 | 80 |
|  |  |  |  |  | 16 | 36 | 17 | 18 | 87 |

**Supplementary Table 4.** Raw scores of the course experience questionnaire for both the control and experimental groups.

| Q1 Work expected | | Q2 Problem-solving skills | | Q3 Analytic skills | | Q4 Memorization of knowledge | | Q5 Motivation | |
| --- | --- | --- | --- | --- | --- | --- | --- | --- | --- |
| Control | Experimental | Control | Experimental | Control | Experimental | Control | Experimental | Control | Experimental |
| 3.5 | 4 | 4.2 | 3.8 | 2.5 | 4.6 | 2.5 | 4.6 | 2.5 | 4.6 |
| 3.4 | 3.8 | 3.2 | 3.5 | 2.6 | 2.5 | 2.6 | 2.5 | 2.1 | 3.5 |
| 3.3 | 4 | 3.8 | 3.6 | 2.7 | 4.2 | 2.7 | 4.2 | 2 | 3.8 |
| 3.6 | 3.6 | 3.4 | 3.1 | 2.4 | 4.3 | 2.4 | 4.3 | 1.9 | 3.6 |
| 3.8 | 4 | 3.2 | 4.2 | 2.5 | 3.8 | 2.5 | 3.8 | 3.8 | 2.8 |
| 3.1 | 3.4 | 3.6 | 4.1 | 3.5 | 3.5 | 3.5 | 3.5 | 3.6 | 5 |
| 2.1 | 4 | 3.2 | 3.5 | 3.4 | 3.6 | 3.4 | 3.6 | 3.4 | 3.6 |
| 2.6 | 3.9 | 4.5 | 3.1 | 3.2 | 3.1 | 3.2 | 3.1 | 3.2 | 3.1 |
| 2 | 4.5 | 4.8 | 3.8 | 3.8 | 4.2 | 3.8 | 4.2 | 3.8 | 4.2 |
| 1.5 | 3 | 2.4 | 4.2 | 3.7 | 2.1 | 3.7 | 2.1 | 3.7 | 3.8 |
| 4.5 | 3.6 | 2.8 | 3.5 | 2.9 | 3.5 | 2.9 | 3.5 | 2.9 | 3.5 |
| 4.3 | 4.2 | 2.5 | 2.9 | 3 | 3.1 | 3 | 4.2 | 3 | 4.2 |
| 3.8 | 3.5 | 2.7 | 3.6 | 1.5 | 3.8 | 1.5 | 4.1 | 2 | 4.1 |
| 1.5 | 3.5 | 3.5 | 4.5 | 3.7 | 4.2 | 4.2 | 3.5 | 4.2 | 3.5 |
| 3.7 | 4.8 | 3 | 5 | 3.5 | 3.5 | 3.2 | 3.1 | 2.5 | 4.6 |
| 3.5 | 3.4 | 2.1 | 3.5 | 3.3 | 2.9 | 3.8 | 3.8 | 2.6 | 4.5 |
| 3.3 | 4.6 | 1.5 | 3.8 | 2.8 | 3.6 | 3.4 | 4.2 | 2.7 | 4.2 |
| 2.8 | 4.8 | 1.9 | 3.6 | 2.5 | 4.5 | 3.2 | 3.5 | 2.4 | 4.3 |
| 2.5 | 4.2 | 3.8 | 2.8 | 2.2 | 5 | 3.6 | 2.9 | 2.5 | 3.8 |
| 2.2 | 4.5 | 3.6 | 5 | 3.4 | 3.5 | 3.5 | 3.6 | 3.5 | 3.5 |
| 3.4 | 4.7 | 3.2 | 3.8 | 3.5 | 4.5 | 3.6 | 4.5 | 3.4 | 3.6 |
| 3.5 | 3.5 | 3.4 | 2.8 | 3.6 | 3.8 | 3.5 | 3.8 | 3.2 | 3.1 |
| 3.6 | 3.1 | 4.2 | 3.4 | 3.5 | 2.7 | 3.1 | 2.7 | 3.8 | 4.2 |
| 4.1 | 3.5 | 4.3 | 4.8 | 3.1 | 3.6 | 3.1 | 3.6 | 3.7 | 4.1 |
|  | 4.2 |  | 3.8 |  | 3.4 |  | 4 |  | 4 |
|  | 3.4 |  | 4.4 |  | 3.5 |  | 3.9 |  | 3.9 |
|  | 3.6 |  | 4.8 |  | 3.5 |  | 4.5 |  | 4.5 |
|  | 3.7 |  | 4.5 |  | 3.8 |  | 3.8 |  | 3.8 |
|  | 4.2 |  | 4.2 |  | 3.4 |  | 3.4 |  | 3.4 |
|  | 4 |  | 4.2 |  | 3.9 |  | 3.9 |  | 3.9 |
| Q6 Pre-class work | | Q7 Helpful feedback | | Q8 Pressure | | Q9 Tackling unfamiliar problems | | Q10 Satisfaction | |
| Control | Experimental | Control | Experimental | Control | Experimental | Control | Experimental | Control | Experimental |
| 2.5 | 2.6 | 2.5 | 4 | 2.5 | 1 | 3.4 | 3.7 | 3.4 | 5 |
| 2.1 | 3.5 | 2.1 | 3.8 | 2.1 | 2.2 | 3.2 | 2.9 | 3.2 | 4.2 |
| 2 | 2.8 | 2 | 4 | 2 | 3 | 3.8 | 3 | 3.8 | 3.5 |
| 1.9 | 2.6 | 1.9 | 3.6 | 1.9 | 1.1 | 3.5 | 4 | 2.5 | 2.9 |
| 3.8 | 2.8 | 3.8 | 4 | 2.5 | 2.3 | 4 | 4.2 | 2.6 | 3.5 |
| 3.6 | 2 | 3.6 | 5 | 2.7 | 1.8 | 2.1 | 2.5 | 2.7 | 2.9 |
| 3.4 | 2.6 | 3.4 | 3.6 | 3 | 1.2 | 3.5 | 3.9 | 2.4 | 3.6 |
| 3.2 | 3.1 | 3.2 | 4.2 | 2.3 | 2.1 | 4.6 | 4.5 | 2.5 | 3 |
| 3.8 | 2.2 | 3.8 | 3.5 | 3 | 1.8 | 3.2 | 3.8 | 3.5 | 3.6 |
| 3.7 | 2.6 | 3.7 | 2.9 | 3.5 | 2.6 | 2.6 | 3.4 | 2.1 | 4.8 |
| 2.9 | 3.5 | 2.9 | 3.6 | 3.1 | 2.6 | 3.5 | 3.8 | 2 | 4 |
| 3 | 2.2 | 3 | 2.5 | 3.2 | 2 | 3.8 | 3.6 | 1.9 | 3.8 |
| 2 | 3 | 2 | 4.1 | 2 | 2.8 | 2.9 | 4.2 | 3.8 | 4 |
| 4.2 | 3.1 | 4.2 | 3.1 | 4.2 | 1.3 | 3.4 | 3.4 | 3.6 | 3.6 |
| 2.5 | 2.3 | 2.5 | 3.8 | 2.5 | 2.8 | 3.1 | 3.2 | 3.7 | 4 |
| 2.6 | 2.4 | 2.6 | 2.2 | 2.6 | 2 | 4 | 3.8 | 4.2 | 5 |
| 2.7 | 2.6 | 2.7 | 3.5 | 2.7 | 2.6 | 2.6 | 2.8 | 3.3 | 4.5 |
| 2.4 | 4 | 2.4 | 2.9 | 1.2 | 2.4 | 2.7 | 4 | 3.4 | 5 |
| 2.5 | 1.8 | 2.5 | 3.6 | 1.5 | 1.4 | 2.4 | 3.6 | 3.2 | 3.1 |
| 3.5 | 3.5 | 3.5 | 4.5 | 1.8 | 2 | 2.5 | 4.5 | 3.8 | 3.8 |
| 3.4 | 4 | 3.4 | 5 | 2.2 | 3.4 | 3.5 | 4.2 | 3.7 | 4.2 |
| 3.2 | 3.1 | 3.2 | 3.1 | 2.1 | 3.2 | 2.1 | 4.3 | 2.9 | 5 |
| 3.8 | 1.6 | 3.8 | 3.8 | 1.9 | 2.8 | 2 | 4.2 | 3 | 3.6 |
| 3.7 | 2.1 | 3.7 | 4.2 | 1.8 | 3.7 | 2 | 4.1 | 2 | 2.5 |
|  | 1.8 |  | 3.5 |  | 3.8 |  | 4 |  | 4.8 |
|  | 1.2 |  | 2.9 |  | 1.6 |  | 3.5 |  | 3.8 |
|  | 2.1 |  | 3.6 |  | 1.3 |  | 3.6 |  | 3.7 |
|  | 2.5 |  | 4.5 |  | 3.2 |  | 4 |  | 3.1 |
|  | 3.4 |  | 3.8 |  | 1.8 |  | 4.2 |  | 3.8 |
|  | 1.2 |  | 3.7 |  | 1.2 |  | 3.8 |  | 2.2 |
